# Chemical profile and antioxidant activity of bidirectional metabolites from *Tremella fuciformis* and *Acanthopanax trifoliatus* as assessed using response surface methodology

**DOI:** 10.3389/fnut.2022.1035788

**Published:** 2022-11-08

**Authors:** Yuxiao Wu, Yu Liu, Jiayi Wu, Kaiqi Ou, Qian Huang, Junxian Cao, Tao Duan, Lin Zhou, Yufang Pan

**Affiliations:** ^1^College of Pharmacy, Guangdong Pharmaceutical University, Guangzhou, China; ^2^Guangdong Province Key Laboratory for Biotechnology Drug Candidates, School of Biosciences and Biopharmaceutics, Guangdong Pharmaceutical University, Guangzhou, China

**Keywords:** *Acanthopanax trifoliatus*, *Tremella fuciformis*, enzymatic hydrolysis, bidirectional fermentation, antioxidant activity, response surface methodology, untargeted metabolomics, phenolic compounds

## Abstract

This study aimed to establish a bidirectional fermentation system using *Tremella fuciformis* and *Acanthopanax trifoliatus* to promote the transformation and utilization of the synthesized antioxidant metabolites from fermentation supernatant. The effect of fermentation conditions on the total phenolic content was investigated using response surface methodology in terms of three factors, including temperature (22–28°C), pH (6–8), and inoculum size (2–8%, v/v). The optimized fermentation parameters were: 28°C, pH 8, and an inoculum size of 2%, which led to a maximum total phenolic content of 314.79 ± 6.89 μg/mL in the fermentation supernatant after 24 h culture. The content of total flavonoids and polysaccharides reached 78.65 ± 0.82 μg/mL and 9358.08 ± 122.96 μg/mL, respectively. In addition, ABTS+, DPPH⋅, and ⋅OH clearance rates reached 95.09, 88.85, and 85.36% at 24 h under optimized conditions, respectively. The content of total phenolics, flavonoids and polysaccharides in the optimized fermentation supernatant of *T. fuciformis*–*Acanthopanax trifoliatus* increased by 0.88 ± 0.04, 0.09 ± 0.02, and 33.84 ± 1.85 times that of aqueous extracts from *A. trifoliatus*, respectively. Simultaneously, 0.30 ± 0.00, 0.26 ± 0.01, and 1.19 ± 0.12 times increase of antioxidant activity against ABTS+, DPPH⋅, and ⋅OH clearance rates were observed, respectively. Additionally, the metabolite composition changes caused by fermentation were analyzed using ultra-high performance liquid chromatography-tandem mass spectrometry (UPLC-MS/MS) based on untargeted metabolomics and the phytochemical profile of fermentation supernatant differentiated significantly based on unsupervised principal component analysis (PCA) during fermentation from 24 to 96 h. Furthermore, a significant increase in antioxidant phenolic and flavonoid compounds, such as ellagic acid, vanillin, luteolin, kaempferol, myricetin, isorhamnetin, and (+)-gallocatechin, was observed after fermentation. Thus, these results indicated that the fermentation broth of *T. fuciformis* and *A. trifoliatus* had significant antioxidant activity, and may have potential application for health products such as functional beverages, cosmetics, and pharmaceutical raw materials.

## Introduction

As one of the secondary metabolites of many species from the plant kingdom, phenolic compounds have become a hot spot in the field of food chemistry and food health due to their unique properties (1). The prevention of cardiovascular and neurodegenerative diseases, diabetes, or obesity due to the consumption of fruits and vegetables has been attributed to their large content of phenolic compounds (2, 3), and may be related to the strong anti-oxidative stress ability of phenolic compounds (4). Inflammation is expected to result in the occurrence of various chronic diseases. Inflammation reaction increases the production of reactive oxygen species (ROS), which have been shown to intensify inflammation (5). Phenolic compounds can inhibit the activity of inflammatory factors, such as cyclooxygenase (COX) and NO synthase (NOS), and downregulate the expression of the transcription factor NF-κB, which is involved in the oxidative stress pathway (6). Therefore, phenolic compounds have the potential to prevent chronic disease.

*Acanthopanax* is a genus of *Araliaceae*, and there are 37 species of *Acanthopanax* plants worldwide. This genus has the reputation of being a “ginseng-like herb” due to the specific active ingredients of Panax (7). *Acanthopanax trifoliatus* is a kind of *Acanthopanax*, also known as Goose’s tendon or trifoliate, that is widely distributed in China, Vietnam, Thailand, Japan, and other countries (8). As a member of this genus with homology for those used in medicine and food, *A. trifoliatus* has functions in traditional Chinese medicine (TCM) including clearing heat and detoxifying, dispelling wind and benefiting dampness, relaxing the body, and promoting blood circulation. In traditional folk therapies, *A. trifoliatus* is often used in the treatment of diabetes, gout, and weak constitutions (9, 10). According to previous studies, *A. trifoliatus* contains a variety of compounds, among which polysaccharides, polyphenols, and volatile oils are the main active components (11). It is worth noting that phenolic compounds from *A. trifoliatus* have strong antioxidant activities (10, 12). In terms of product application, the leaves of *A. trifoliatus* are often used, such as in high-quality teas from the Guangdong province of China, although the production of tea leads to the waste of rhizomes and other parts of this plant. In this paper, we utilized the stem of *A. trifoliatus* as a research object to explore the possibility of expanding the uses of this plant in new applications.

*Tremella fuciformis* is the fruiting body of a fungus of the genus *Tremella*, also known as white fungus. It has high nutritional value and can also be used for both medicines and in food, and it is known as the “crown of fungi.” Studies have shown that *T. fuciformis* exhibits unique biological activities and has a positive regulatory effect on inflammation, tumor growth, hyperglycemia, and other diseases (13–15). As is well known, *T. fuciformis* polysaccharides are among the most studied and widely used active ingredient from *T. fuciformis* (15, 16). Some studies have indicated that the phenolic acids, catechins, flavonoids, and other active ingredients of *T. fuciformis* have strong antioxidant and anti-inflammatory effects (17–19). However, the phenolic compounds from *T. fuciformis* are often underestimated in practical applications due to their relatively low concentrations. Based on this, it seems that there is a good prospect to expand research on *T. fuciformis*.

Bidirectional fermentation refers to a situation when a preferred medicinal bacteria is used as a fermentation strain, and TCM materials or medicinal residues with certain active ingredients are used as fermentation substrates instead of traditional nutrients. The advantages of bidirectional fermentation are that it better promotes the extraction of active ingredients, increases the efficacy of fermentation, and can even produce new active substances (20). In our recent work, exopolysaccharide yields and antioxidant activities were significantly improved using *Schizophyllum commune* fermented products by bidirectional fermentation with *Radix Puerariae* (21), and these metabolites could extend the lifespan and health-span of *Caenorhabditis elegans* (22).

It is well known that cellulase can degrade plant fibrous tissue, causing cell rupture and efflux of intracellular nutrients during fermentation (23). Therefore, the extraction rate of active ingredients can be improved using cellulose (24). It should be noted that *T. fuciformis* has little ability to decompose cellulose, and needs to cooperate with extracellular enzymes from companion fungi or other enzymes (25). The combination of enzymatic pre-treatment before bidirectional fermentation may promote the synthesis of more antioxidant metabolites, which is also discussed in this work. Thus, liquid fermentation was carried out with a stem of *A. trifoliatus* pre-treated with cellulase as a fermentation substrate and *T. fuciformis* as an additive fermented strain, which could not only make full use of plant resources but also produce valuable active ingredients. The fermentation products with high content of phytochemical and strong antioxidant capacities were generated by optimizing fermentation conditions. Furthermore, untargeted metabolomics analysis using ultra-high performance liquid chromatography-tandem mass spectrometry (UPLC-MS/MS) was used to elucidate the changes in active phenolic components during fermentation.

## Materials and methods

### Materials and chemicals

*Tremella fuciformis* was obtained from Sanming Mycology Research Institute of Fujian province, and *A. trifoliatus*, which was identified by Associate Professor Liu Jizhu of Guangdong Pharmaceutical University as the *Araliaceae* plant (*A. trifoliatus (L.) Merr*), was purchased from Enping, Guangdong. These specimens were preserved in the Herbarium of the School of Traditional Chinese Medicine, Guangdong Pharmaceutical University. Cellulase, Folin phenol, ABTS, and DPPH were purchased from McLean Technology Co., Ltd. (Guangdong, China) Gallic acid and rutin were purchased from Yuanye Biotechnology Co., Ltd. (Shanghai, China). Sodium nitrite, salicylic acid, glucose, and potassium persulfate were purchased from Zhiyuan Chemical Reagent Co., Ltd. (Tianjin, China). Liquid chromatograph mass spectrometer (LC-MS)-grade methanol (MeOH) was purchased from Fisher Scientific (Loughborough, UK). The 2-chloro-L-phenylalanine was obtained from Aladdin (Shanghai, China). LC-MS-grade acetonitrile (ACN) was purchased from Fisher Scientific (Loughborough, UK). Formic acid was obtained from TCI (Shanghai, China). Ammonium formate was obtained from Sigma-Aldrich (Shanghai, China). All other reagents and chemicals were of analytical grade.

### Material pre-treatment and fermentation procedures

The stems of *A. trifoliatus* were collected and cut into 2-cm pieces, pulverized using a pulveriser (YB-1000A, Yun bang, Zhejiang, china), and sieved through a 35-mesh sieve to obtain an *A. trifoliatus* fine powder. After distilled water at a solid-liquid mass ratio of 1: 20 (v/v) was added to the fine powder, the solution containing the medicinal powder was kept in a boiling water bath (HHS-2S, Yichang, Shanghai, China) at 100°C for 2 h. Aqueous extracts were then collected using a vacuum filtration device (power of 180 W, voltage of 220 v/50 Hz, and flow rate of 60 L/min) (SHZ-III, Yarong, Shanghai, China), then the same volume of distilled water was added to the powder and the boiling water bath extraction and collection procedure was repeated. The two aqueous extracts were then mixed, and aqueous extracts of *A. trifoliatus* (AE-AT) were finally obtained (26).

The method described by Tao et al. (27) was slightly modified, and AE-AT was enzymatically pre-treated with cellulase. Cellulase buffer was prepared at a mass ratio of 1: 85 (v/v) using PBS phosphate buffer solution (pH = 4.8). The pH of AE-AT was adjusted to the optimum working pH of 4.80 for cellulase using hydrochloric acid (4.5%, v/v). After AE-AT and cellulase buffer were separately pre-heated in a 55°C water bath (HHS-2S, Yichang, Shanghai, China) for 10 min, the enzyme solution (1.2% V/V) was added to AE-AT and continually reacted in a water bath (HHS-2S, Yichang, Shanghai, China) at 55°C for 2 h. The final enzymatic hydrolysis solution was incubated in a boiling water bath (HHS-2S, Yichang, Shanghai, China) for 10 min to inactivate cellulase in the hydrolysis solution. After standing and cooling to room temperature, the enzymatic hydrolyzate was filtered with Whatman NO.1 filter paper and finally enzymatic hydrolyzate *A. trifoliatus* (EH-AT) was prepared.

#### Strain activation

A small amount of marginal hyphae from *T. fuciformis* stored at 4°C was scraped and inoculated onto a solid medium plate commonly used for bacterial activation, passaged and the colony characteristics were observed, followed by activation at 25°C for 4 days, and the above passage procedure was repeated once. Then, the strains were inoculated into a liquid culture flask as the bacteria and nutrients in the liquid medium will be fully mixed, which was grown rapidly and was used for fermentation experiments. Furthermore, each flask was incubated in a shaking incubator (ZQPW-70, Labotery, Tianjin, China) at 25°C and 120 r/min for 4 days.

#### Supplementary medium

During this step, 8 g glucose, 0.4 g MgSO_4_⋅7H_2_O, 0.2 g KH_2_PO_4_, and 2.0 g yeast extract were dissolved in 400 mL of EH-AT solution. The pH of the EH-AT solution was adjusted for one-factor experiments or to the response surface experimental setpoint and then divided into 100-mL Erlenmeyer flasks, which were then sterilized at a high temperature of 121°C for 25 min.

#### Fermentation procedure

*Tremella fuciformis* in the liquid medium was first inoculated into EH-AT solution. After culture for 96 h, the obtained fermentation broth was filtered with eight layers of gauze because different specifications of filter paper easily block the fine mycelium of *T. fuciformis*. The mycelia were removed using centrifugation at 16,099 × *g* for 10 min to obtain the fermentation supernatant of *T. fuciformis*–*A. trifoliatus* (TF-AT). A 10 mL sample of fermentation broth was taken every 24 h and the supernatant was prepared using centrifugation at 16,099 × *g* for 10 min, with the supernatant solution finally being stored at −20°C until analysis.

### Design of single-factor experiments

Three regular factors for different inoculum amounts (2, 5, 8, and 11%, v/v), temperature (22, 25, 28, and 31°C), pH (5, 6, 7, and 8) were selected to increase the phenolic content. While one of the variables was being evaluated, the other fixed parameters were set to an inoculum size of 5% (v/v), a temperature of 25°C, and an initial pH of 6.0. The results of the single factor screening were then used as a reference for the response surface.

### Design and optimization of response surface methodology

A Box–Behnken design (BBD) in response surface methodology (RSM) was selected to determine the optimal fermentation conditions for TF-AT. Based on the principles of BBD experimental design and the data obtained from our single-factor experiments, the total phenolic content of the fermentation broth was selected as the response value. Three factors (A of temperature, B of inoculum, and C of pH) that had a significant impact on the total phenolic content of the fermentation broth were selected as dependent variables to optimize the process parameters of this fermentation system. Table 1 presents the factor and level design.

**TABLE 1:** Factors and levels of the Box–Behnken design.

Levels	Factors		
	A: Fermentation temperatures (°C)	B: Inoculum size (%)	Initial pH
−1	22	2	6
0	25	5	7
1	28	8	8

### Determination of total phenolics

The Folin–Ciocalteu colorimetric method was used to measure the total phenolic content in fermentation broth according to the method described in Darikvand et al. (28) with minor modification. Briefly, 0.1 mL of the sample solution was mixed with 0.1 mL of Folin–Ciocalteu reagent. The resulting mixture was left at room temperature for 6 min. Then, 1 mL of 7% (v/v) sodium carbonate solution and 0.8 mL of pure water were added to the mixture. After mixing, the reaction mixture was kept at room temperature for 60 min in the dark. Finally, the resulting mixture was measured at 510 nm, and gallic acid (50–300 μg/L) was used as a standard to quantify the total phenolic content.

### Determination of total flavonoids

Total flavonoid content was determined according to the protocol described in Gautam et al. (29) with minor modifications. In brief, 1 mL of fermentation supernatant and 150 μL of sodium nitrite 5% (v/v) were added to a 5-mL test tube. The mixture was allowed to stand for 6 min. Then, 150 μL of 10% (v/v) aluminum chloride solution was added to each tube, mixed and incubated for 6 min. Two milliliters of a 4% (v/v) sodium hydroxide solution was added to the test tube. Finally, absolute ethanol was added to each tube to bring the final volume to 5 mL and reactions proceeded at room temperature for 15 min in the dark. Each mixture was measured at 510 nm, and rutin (50–250 μg/mL) was used as a standard to quantify the total flavonoid content.

### Determination of total polysaccharides

One mL of a phenol solution at a concentration of 6% (v/v) was added to 1 mL of fermentation supernatant. After vortexing, 5 mL of concentrated sulfuric acid was added to each mixture, which were then vortexed again, and then left to stand at room temperature for 30 min. Glucose (50–500 μg/mL) was used as a standard to calculate total sugar content.

Additionally, the above method was used to determine the total polysaccharide content, because glucose was added when supplementing the fermentation medium, and reducing sugars can also be produced during fermentation. Then the DNS method (30) for the determination of reducing sugars was used. After the reducing sugars were subtracted from the total sugar content, the total polysaccharide content was determined.

### Antioxidant activity assays

#### Assay of ABTS+ radical scavenging capacity

The method described in Deng et al. (21) was used with simple modifications. ABTS+ reagent was prepared as follows: 1.5 mL of 7 mM ABTS+ solution was added to 0.75 mL of 7.35 mM potassium persulfate solution, mixed at a ratio of 2:1 (v/v), and the mixture was placed in the dark for 16 h. Each mixture was then diluted with phosphate buffer until the absorbance value reached 0.70 ± 0.02 before use.

Sample measurement: 50 μL of each sample was added to 3 mL of ABTS+ free radical solution, mixed well, and placed in the dark for 8 min. Each mixture was measured at 734 nm, distilled water was used as a blank control, and ascorbic acid was used as a positive control. Equation 1 was used to calculate the ABTS+ scavenging activity of each sample:


(1) $$\mathrm{ABTS}+\mathrm{scavenging}\mathrm{activity}\left(\%\right)=\left[1-\frac{{\text{A}}_{1}-{\text{A}}_{2}}{{\text{A}}_{0}}\right]\times 100\%$$

where the absorbance value of a sample when ABTS+ was added is A_1_, the absorbance value when the ABTS+ solution was replaced by an equal volume of phosphate buffer is A_2_, and the absorbance value when the sample was replaced by an equal volume of phosphate buffer is A_0_.

#### Assay of DPPH⋅ radical scavenging capacity

The method described in Chen et al. (31) was used with minor modification. Briefly, 50 μL of fermentation supernatant was collected in a 5 mL brown centrifuge tube, and then 1.5 mL of 0.07 mg/mL DPPH⋅ was added to each centrifuge tube, the samples were mixed, and then placed in the dark for 30 min. Absolute ethanol was used as a blank control and ascorbic acid was used as a positive control. Each sample was measured in triplicate, then the resulting mixture was measured at 517 nm. Equation 2 was used to calculate the DPPH⋅ scavenging activity of each sample:


(2) $$\mathrm{DPPH}\cdot \mathrm{scavenging}\mathrm{activity}\left(\%\right)=\left[1-\frac{{\text{A}}_{\text{i}}-{\text{A}}_{\text{u}}}{{\text{A}}_{\text{z}}}\right]\times 100\%$$

where the absorbance value is A_*i*_ when the sample was added to DPPH⋅, the absorbance value is A_*u*_ when the DPPH⋅ solution is replaced by 3 mL of anhydrous ethanol, and the absorbance value is A_*Z*_ when the sample is replaced by an equal volume of anhydrous ethanol.

#### Hydroxyl radical scavenging activity

Using the method in Li et al. (32), 0.5 mL of fermentation supernatant was added to a mixture of 1 mL of 6 mM ferrous sulfate and 1 mL of 6 mM salicylic acid-ethanol. The mixture was mixed and then left to stand for 10 min. 0.5 mL of 8.8 mM hydrogen peroxide was then added to each mixture and samples were allowed to stand in the dark for 30 min. Each mixture was measured at 517 nm, and VC was used as a positive control. Equation 3 was used to calculate the OH radical scavenging activity of each sample:


(3) $$\cdot \mathrm{OH}\mathrm{scavenging}\mathrm{activity}\left(\%\right)=\left[1-\frac{{\text{A}}_{\text{b}}-{\text{A}}_{\text{c}}}{{\text{A}}_{\text{d}}}\right]\times 100\%$$

where the absorbance value is A_*b*_ when the sample is added, the absorbance value when H_2_O_2_ is replaced by an equal volume of distilled water is A_*c*_, and the absorbance value when the sample is replaced by an equal volume of distilled water is A_*d*_.

### Analysis of untargeted metabolomics using ultra-high performance liquid chromatography-tandem mass spectrometry

#### Sample preparation

Experimental samples were thawed at 4°C, and the thawed samples were vortexed for 1 min. Two milliliters of each sample was pipetted into a centrifuge tube and concentrated to dryness. 500 μL of a methanol solution (stored at −20°C) was added to each centrifuge tube, and after 1 min of vortexing, each sample was centrifuged at 16,099 × *g* at 4°C for 10 min. The supernatant was completely removed and transferred to a new 2-mL centrifuge tube and then concentrated to dryness again. Next, 150 μL of a 2-chloro-L-phenylalanine (4 ppm) solution prepared with 80% methanol-aqueous (stored at −20°C) was added to each centrifuge tube to reconstitute each sample. The resulting supernatant was filtered through a 0.22-μm membrane, and the filtrate was added to a detection bottle as a sample to be tested using UPLC-MS/MS (33).

#### Liquid chromatography and mass spectrometry conditions

Liquid chromatograph analysis was performed on a Vanquish UHPLC System (Thermo Fisher Scientific, Waltham, MA, USA). Chromatography was carried out with an ACQUITY UPLC^®^ HSS T3 (150 × 2.1 mm, 1.8 μm) (Waters, Milford, MA, USA). The column was maintained at 40°C. The flow rate and injection volume were set at 0.25 mL/min and 2 μL, respectively. For LC-ESI (+)-MS analysis, the mobile phases consisted of (B2) 0.1% formic acid in acetonitrile (v/v) and (A2) 0.1% formic acid in water (v/v). Separation was conducted under the following gradient: 0–1 min, 2% B2; 1–9 min, 2–50% B2; 9–12 min, 50–98% B2; 12–13.5 min, 98% B2; 13.5–14 min, 98–2% B2; and 14–20 min, 2% B2. For LC-ESI (-)-MS analysis, the analytes were (B3) acetonitrile and (A3) ammonium formate (5 mM). Separation was conducted using the following gradient: 0–1 min, 2% B3; 1–9 min, 2–50% B3; 9–12 min, 50–98% B3; 12–13.5 min, 98% B3; 13.5–14 min, 98–2% B3; and 14–17 min, 2% B3 (34).

Mass spectrometric detection of metabolites was performed on an Q Exactive Focus instrument (Thermo Fisher Scientific, Waltham, MA, USA) with an ESI ion source. Simultaneous MS1 and MS/MS (Full MS-ddMS2 mode, data-dependent MS/MS) acquisition was used. The parameters were as follows: sheath gas pressure, 30 arb; aux gas flow, 10 arb; spray voltage, 3.50 and −2.50 kV for ESI (+) and ESI (−), respectively; capillary temperature, 325°C; MS1 range, m/z 100–1000; MS1 resolving power, 70000 FWHM; the number of data dependant scans per cycle, 3; MS/MS resolving power, 17500 FWHM; normalized collision energy, 30 eV; dynamic exclusion time, automatic (35).

### Statistical analyses

Statistical analysis was performed using IBM SPSS 20.0. Significant differences between groups were determined using a one-way analysis of variance (ANOVA). *p* < 0.05 was considered statistically significant. Untargeted metabolomic analysis was performed by wekemo bioincloud on the website of https://www.bioincloud.tech/.

## Results and discussion

### Variation in the total phenolic content of the fermentation supernatant of *Tremella fuciformis*–*Acanthopanax trifoliatus* in single factor experiments

#### Effects of different inoculum sizes

**FIGURE 1:**
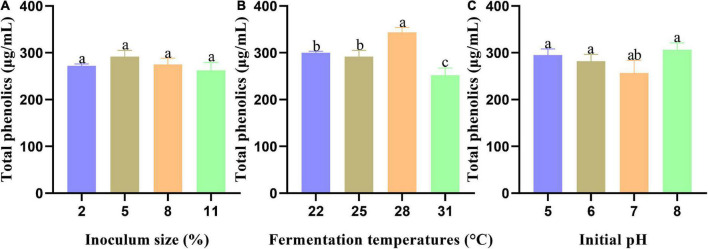
The effect of every single factor on the total phenolic content of fermentation broth: **(A)** Inoculum size, **(B)** Fermentation temperatures, and **(C)** Initial pH. Data are expressed as mean ± SD, *n* = 3. Values with no letters in common are significantly different (*p* < 0.05).

The range from 2 to 11% (v/v) was selected to explore the effect of inoculum size on total phenolic content, while other fixed parameters were set as a temperature of 25°C, and an initial pH of 6.0. As shown in Figure 1A, with the increase in the inoculation amount, the total phenolic content of the fermentation broth showed an overall trend of increasing first and then decreasing without significance (*p* > 0.05). The total phenolic content reached a maximum value of 291.93 ± 13.11 μg/mL with a 5% inoculum size. Ojo et al. (36) found that the total phenolic content increased with increasing inoculum size (5–15%, v/v) of *Rhizopus oligosporus* during solid-state fermentation of cassava stems. The total phenolic content decreased gradually when the inoculation amount was larger than 5% (v/v). A lower level of 262.17 ± 17.04 μg/mL was shown at the inoculum amount of 11% (v/v). As it is important to achieve a balance between inoculum size and available nutrients, too high of an inoculum size might result in the nutrient competition of strains on limited substrates (37). Here, an inoculum size of 2–8% (v/v) was selected for further response surface analysis.

#### Effects of different fermentation temperatures

A range from 22 to 31°C was chosen to study the effects of temperature on the total phenolic content, while other parameters were fixed, including an inoculum volume of 5% (v/v), and an initial pH of 6.0. As shown in Figure 1B, when the temperature reached 28°C, the total phenolic content of the fermentation broth reached its highest level of 343.60 ± 10.26 μg/mL, which was significantly different from the three other temperature gradients (*p* < 0.05). The total phenolic content showed a downward trend at 31°C, which might be related to the optimum growth temperature of *T. fuciformis* (38). Considering that the growth of *T. fuciformis* will be inhibited by a high temperature, 22–28°C was selected for subsequent analyses.

#### Effects of initial pH in culture medium

An initial pH range from 5 to 8 was chosen to investigate the effects of pH on the total phenolic content. An inoculum volume of 5% (v/v), and a temperature of 25°C were used as fixed parameters. As displayed in Figure 1C, with an increase in initial pH, the total phenolic content of the fermentation broth showed a trend of decreasing first and then increasing, reaching a peak value of 306.45 ± 14.67 μg/mL at pH 8. Cho et al. (39). showed that maximum cell growth and exopolysaccharide content formation were achieved at an alkaline pH of 8.0–9.0 in a submerged culture of *T. fuciformis*. It was also speculated that weak alkali conditions were suitable for the growth of *T. fuciformis*, which was closely related to the total phenolic yield of the fermentation broth in this study. Considering the comprehensive experimental results, pH values from 6 to 8 were selected for additional analyses.

It should be noted that no significant differences were observed of the pH, inoculum size and temperature in our test even though we select wide ranges for these parameters, such as an inoculum size from 2 to 11% (v/v). However, complex interactions could possibly exist between these parameters, which was addressed by the following RSM study. One possible reason for the insignificant univariate analysis was total content of polyphenol content changed only slightly during 24–96 h culture (Figure 3), but the varieties of polyphenols changed significantly, which was consistent with our subsequent analysis of untargeted metabolomics results.

**FIGURE 2:**
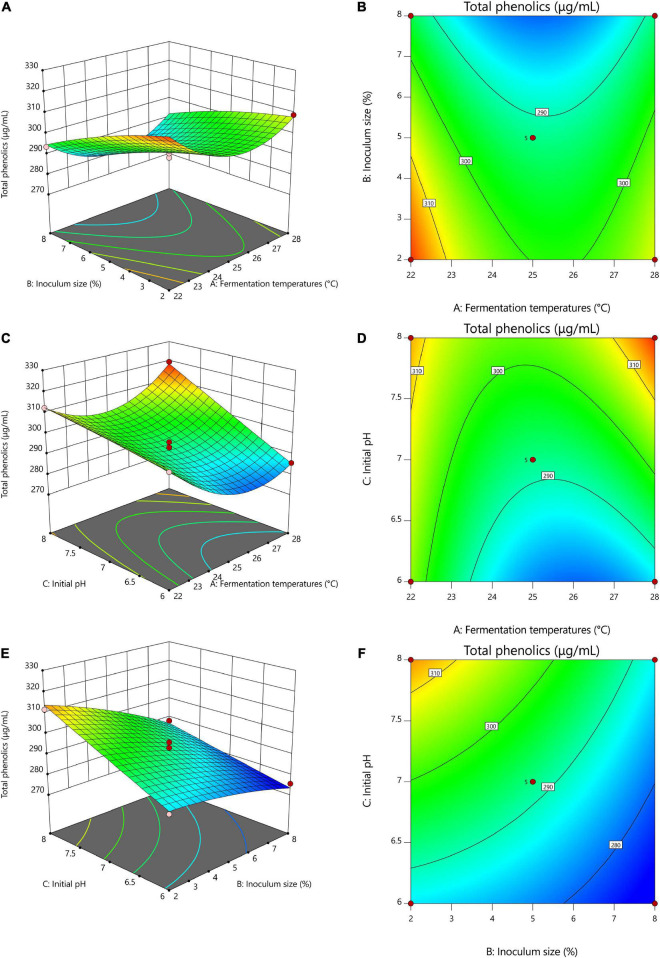
Response surface optimization of total phenolics with different fermentation conditions. **(A,B)** 3D and contour plots of the interaction between fermentation temperatures and inoculum size, **(C,D)** 3D and contour plots of the interaction of fermentation temperatures with initial pH, **(E,F)** 3D and contour plots of the interaction of inoculum size with initial pH. **(A)** Fermentation temperatures, **(B)** Inoculum size, **(C)** Initial pH.

**FIGURE 3:**
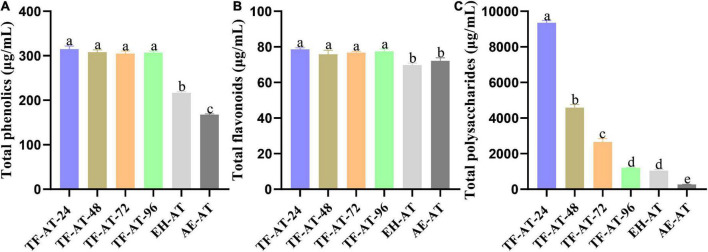
Content comparison of **(A)** total phenolics, **(B)** total flavonoids, and **(C)** total polysaccharides among different groups. Data are expressed as mean ± SD, *n* = 3. Values with no letters in common are significantly different (*p* < 0.05). AE-AT: *aqueous extracts*–*Acanthopanax trifoliatus*; EH-AT: *enzymatic hydrolyzate*–*Acanthopanax trifoliatus*; TF-AT: fermentation supernatant of *Tremella fuciformis*–*Acanthopanax trifoliatus*. TF-AT-24, 48, 72, and 96 mean samples with related fermentation time (h).

### Response surface methodology optimization results and analysis

#### Response surface methodology model linear fitting analysis

Table 2 displays the response surface optimization test plan and results. Multiple regression fitting was analyzed using Design Expert 11, and a multivariate quadratic regression model equation between (A) fermentation temperatures, (B) inoculum size, (C) initial pH, and the response value (total phenol content) was obtained as (4):


Total⁢phenolics⁢(μ⁢g/mL)= 291.98-3.18⁢A-9.88⁢B+

$$10.33\,\text{C}+1.78\,\text{AB}+6.13\,\text{AC}-3.57\,\text{BC}+13.16\,{\text{A}}^{2}-2.02\,{\text{B}}^{2}$$

(4) $$+0.065\,{\text{C}}^{2}$$

**TABLE 2:** Box–Behnken design matrix used in RSM with experimental responses.

Run	A: Fermentation temperatures (°C)	B: Inoculum size (%)	C: Initial pH	Total phenolics (μg/mL)
1	−1	−1	0	320.26
2	1	−1	0	309.07
3	−1	1	0	293.60
4	1	1	0	289.55
5	−1	0	−1	303.12
6	1	0	−1	285.74
7	−1	0	1	312.41
8	1	0	1	319.55
9	0	−1	−1	284.79
10	0	1	−1	275.50
11	0	−1	1	311.69
12	0	1	1	288.12
13	0	0	0	288.36
14	0	0	0	290.50
15	0	0	0	295.98
16	0	0	0	293.36
17	0	0	0	291.69

where A = Fermentation temperature, B = Inoculum size, and C = Initial pH. The equation of this established model showed that factor C was positively correlated with the total phenolic content of the fermentation supernatant, while factors A and B were negatively correlated.

As shown in Table 3, *R*^2^ and Adj*R*^2^ were 0.9779 and 0.9496, respectively, both of which were close to 1, indicating that the model correlation was good. The signal-to-noise ratio obtained using Adeq Precision = 20.0481 (>4), indicating that the model was relatively undisturbed by external factors. Moreover, the C.V.% = 0.9865, indicating that the model had a very small degree of dispersion. The comprehensive situation showed that the model had high reliability and strong stability, and could be used for simulation prediction of fermentation test results.

**TABLE 3:** Analysis of variance (ANOVA) for Box–Behnken design.

Sources	Sum of squares	*df*	Mean square	*F*-value	*P*-value	
Model	2667.78	9	296.42	34.47	<0.0001	***
A	81.09	1	81.09	9.43	0.0180	*
B	780.92	1	781.92	90.82	<0.0001	***
C	853.05	1	853.05	99.21	<0.0001	***
AB	12.74	1	12.74	1.48	0.2629	−
AC	150.43	1	150.43	17.49	0.0041	***
BC	50.98	1	50.98	5.93	0.0451	*
A^2^	729.17	1	729.17	84.80	<0.0001	***
B^2^	17.14	1	17.14	1.99	0.2008	−
C^2^	0.0177	1	0.0177	0.0021	0.9651	−
Residual	60.19	7	8.60	−	−	−
Lack of Fit	26.91	3	8.97	1.08	0.4532	−
Pure Error	33.28	4	8.32	−	−	−
Cor Total	2727.97	16	−	−	−	−
*R*^2^ = 0.9779 Adj*R*^2^ = 0.9496 Adeq Precision = 20.0481 C.V.% = 0.9865						

*Significant at 0.01 < *p* < 0.05, and ***significant at *p* < 0.001.

#### Model interaction analysis

Table 3 also demonstrates that our model, with an *F* = 34.47 and *p* < 0.001, was extremely significant. The lack of fit items with *F* = 1.08 and *p* > 0.05 was not significant, indicating that the model fit well. Our precise analysis and prediction of the total phenolic content of the fermentation broth showed that the results were statistically significant. Judging from the *F*-value, the order of the influence of every single factor on the total phenolic content was as follows: (C) Initial pH > (B) Inoculum size > (A) Fermentation temperatures. The order of the influence of the interaction factor on the total phenolic content was: AC > BC > AB. The order of the influence of the quadratic factor on the total phenolic content was A^2^ > B^2^ > C^2^.

Figure 2 shows the 3D and contour maps of the response surface of the RSM. The effect of two interaction variables on the total phenolic content of TF-AT can be observed from these two maps. From the effect of the interaction between fermentation temperatures and inoculum size on the total phenolic content in Figures 2A,B, with increasing inoculum amount (2–8%, v/v), the total phenolic content decreased significantly (from 320.26 to 293.60 μg/mL), while the total phenolic content presented a trend of decline first and then increase from low to high temperature (22–28°C). Overall, the AB interaction graph was relatively flat, and the contour graph was approximately elliptic, but the overall effect on total phenolic content was not significant, indicating that the interaction between fermentation temperatures and inoculum size was weak. Similarly, Figures 2C,D illustrate that with an increase of pH from 6 to 8, the total phenol content increased (303.12–312.41 μg/mL). The slope of the 3D map was steep, and the contour map was approximately elliptical, indicating that the interaction between fermentation temperatures and initial pH was strong. Furthermore, the overall effect on the total phenolic content was significant (*p* < 0.05), which was consistent with the results of variance analysis. Figures 2E,F were relatively flat. The contour lines tended to be circular, and the interaction between the two variables was weak. In conclusion, these results indicated that fermentation temperatures, inoculum size, and initial pH had critical effects on total phenolic content.

#### Optimal fermentation processes

Our model predicts that the optimal conditions for TF-AT fermentation were a fermentation temperature of 27.78°C, an inoculum size of 2.52%, and an initial pH of 7.84, resulting in the predicted 321.70 μg/mL of total phenolics in the fermentation broth. For the controllability of the actual fermentation parameters, the optimal conditions were adjusted to fermentation temperatures of 28°C, and inoculum size of 2%, and initial pH of 8. Under these conditions, the total phenolic content was 305.02 μg/mL, and the error value from the predicted value was 5.5%. The results showed that the model obtained in this test was feasible.

### Bioactive components of *Tremella fuciformis*–*Acanthopanax trifoliatus* under optimal fermentation conditions

#### Analysis of total phenolic content

To further explore the effects of adding cellulase and *T. fuciformis* on the total phenolic content of AE-AT, the total phenolic content of AE-AT, EH-AT, and the fermentation supernatant from TF-AT were measured, respectively. TF-AT was divided into four groups (24, 48, 72, and 96 h) in chronological order. Figure 3A shows that the total phenolic content of AE-AT was at a low level of 167.88 ± 1.49 μg/mL. The total phenolic content of the EH-AT group with cellulase added was significantly improved (*p* < 0.01) to 216.93 ± 2.14 μg/mL, which increased 0.29 ± 0.00 times that of AE-AT. It is worth noting that in the TF-AT group supplemented with *T. fuciformis*, the total phenolic content was also significantly increased (*p* < 0.01). The total phenolic content of TF-AT-24 fermented for 24 h (314.79 ± 6.89 μg/mL) increased 0.45 ± 0.03 and 0.88 ± 0.04 times compared with that from EH-AT and AE-AT, respectively.

Previous studies have shown that phenolic compounds usually exist in various plants in the form of hydroxyl groups and sugars or glycosides through conjugation (40). In addition, β-glucosidase, α-amylase, cellulase, among others, could effectively eliminate ether bonds between phenolic compounds and cell walls (41, 42). According to Juan et al. (43), in the solid-state fermentation of black soybean, *Bacillus subtilis BCRC 14715* produced a substance of β-glucosidase that acts to hydrolyze the ether bond, thereby releasing more phenolic compounds. Xin et al. (44) studied the wood flour degradation ability of *T. fuciformis* and its associated cellulase and found that they had a strong synergistic effect, and the degradation ability was significantly improved by 5-fold. Interestingly, Zhang et al. (45) found that the total phenolic content obtained from the co-fermentation of compound enzymes and lactic acid bacteria was much higher than that obtained by single enzyme decomposition. These results were similar to this study, that is, the total phenolic content of EH-AT obtained by enzymatic pre-treatment was higher than that of AE-AT, and the total phenolic content of TF-AT obtained by the combined action of enzymatic hydrolysis and *T. fuciformis* was much higher than that of AE-AT, showing a strong ability to enrich biological activity. In addition, cellulose is the main carbon source in the biosphere, and after being degraded by cellulase, the carbon source in it will be exposed and used by microorganisms (46).

#### Analysis of total flavonoid content

To ensure the uniformity of our experiments, we measured the flavonoid content of AE-AT, EH-AT, and the fermentation supernatant from TF-AT, respectively. Figure 3B shows that the flavonoid content of EH-AT was 69.79 ± 1.08 μg/mL, while the flavonoid content of AE-AT reached 72.12 ± 1.79 μg/mL but with no significantly increase (*p* > 0.05) compared to the EH-AT group. However, the flavonoid content of TF-AT during 24–96 h culture all increased significantly (*p* < 0.05) compared to the AE-AT and EH-AT groups. In particular, the flavonoid content of the fermentation supernatant from TF-AT-24 obtained after 24 h culture reached 78.65 ± 0.82 μg/mL, which increased by 0.13 ± 0.02 and 0.09 ± 0.02 times that of EH-AT and AE-AT, respectively.

It is generally known that flavonoids in plants belong to the category of polyphenolic compounds, which also have a wide range of biological activities, and their benefits to human health should not be underestimated. In previous studies, more than 9,000 flavonoids have been discovered, which are highly exploitable (47, 48). Usually, when the ether bond between a bioactive ingredient and the cell wall is broken by cellulase, and many bioactive components leave a cell, the changes in flavonoid content and total phenolic content were consistent with the same upward trend (39, 40). Studies have shown that flavonoids are prone to oxidative decomposition (49) and are easily degraded or polymerized by certain oxidative enzymes (50). Additionally, compared to the solely cellulase degradation, cellulase degradation coupled with *T. fuciformis* fermentation showed much stronger increases in the promoting effect on the total flavonoid content in current study.

#### Analysis of total polysaccharide content

The polysaccharides of *A. trifoliatus* and *T. fuciformis* are important bioactive components, and have positive nutritional and therapeutic effects on diseases such as diabetes and hypertension (10, 15). Therefore, to ensure the integrity of our experiment, the total polysaccharide content in different groups was analyzed. Figure 3C indicates that the total polysaccharide content of the fermentation supernatant of TF-AT was very significantly increased (*p* < 0.01) at 24 h, reaching 9358.08 ± 122.96 μg/mL, which increase 33.84 ± 1.85 and 7.94 ± 0.61 times that of AE-AT and EH-AT, respectively. Fungi or enzymes can produce different carbohydrate enzymes that allow the cellulosic cell walls of plants or fruits and vegetables to be broken down into soluble polysaccharides, increasing the permeability of the cell walls and promoting the efflux of polysaccharides (51). However, as the fermentation progresses, polysaccharides may also be broken down into monosaccharides or oligosaccharides by various enzymes, and finally metabolized to short-chain fatty acids (52), which may explain why the total polysaccharide content of the fermentation supernatant of TF-AT showed a significant downward trend (*p* < 0.01) as the fermentation proceeded, and finally reached the low level of 1203.95 ± 11.47 μg/mL at 96 h, which was almost the same as that of EH-AT. However, further research is needed to infer the changing law of polysaccharide content and their chemical structures during the bidirectional fermentation of *A. trifoliatus* and *T. fuciformis*.

### Analysis of antioxidant capacity

**FIGURE 4:**
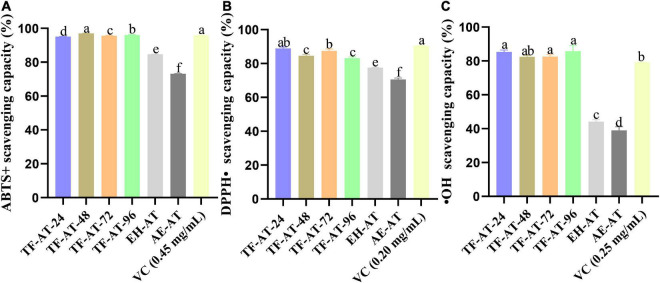
*In vitro* antioxidant activity among different groups, **(A)** ABTS+ scavenging capacity, **(B)** DPPH⋅ scavenging capacity, and **(C)** ⋅OH scavenging capacity. Data are expressed as mean ± SD, *n* = 3. Values with no letters in common are significantly different (*p* < 0.05). AE-AT: *aqueous extracts*–*Acanthopanax trifoliatus*; EH-AT: *enzymatic hydrolyzate*–*Acanthopanax trifoliatus*; TF-AT: fermentation supernatant of *Tremella fuciformis*–*Acanthopanax trifoliatus.* TF-AT-24, 48, 72, and 96 mean samples with related fermentation time (h).

The fermentation supernatant of TF-AT can increase a variety of effective active ingredients. Many studies have shown that fermentation not only increases the active components but also increases the scavenging ability of free radicals (53). Different antioxidant assays were thus used to verify whether fermented TF-AT increased antioxidant capacity. Our scavenging experiments targeting free radicals such as ABTS+, DPPH⋅, and ⋅OH have been applied widely for *in vitro* antioxidant capacity determination. Figures 4A–C present the scavenging effects of these three free radicals, respectively. The ABTS+ clearance rate of AE-AT (Figure 4A) was 73.18%, while the EH-AT clearance rate obtained by adding cellulase enzymatic pre-treatment increased to 84.73%. After fermentation, the clearance rate of the fermentation supernatant of TF-AT at 24 h reached 95.09%, which increased for 0.12 ± 0.00 and 0.30 ± 0.00 times compared with EH-AT and AE-AT, respectively. The ABTS+ clearance ability of the TF-AT-24 sample was comparable to the positive control ascorbic acid at 0.45 mg/mL. With increasing fermentation time, TF-AT still showed a very high ABTS+ scavenging ability. Figure 4B shows the results of the DPPH⋅ free radical scavenging rate. The DPPH⋅ scavenging rate of AE-AT is 70.52%, while that of EH-AT has increased to 77.54%. The addition of *T. fuciformis* signaled the beginning of fermentation, the DPPH⋅ clearance rate reached 88.85% (take 24 h as an example), which increased for 0.15 ± 0.01 and 0.26 ± 0.01 times compared with EH-AT and AE-AT, respectively. The DPPH⋅ clearance ability of the TF-AT-24 sample was comparable to the positive control ascorbic acid at 0.20 mg/mL.

It can be seen from Figure 4C that the scavenging capacity of OH radicals of AE-AT and EH-AT were 39.05 and 44.24%, respectively. However, the OH radical scavenging ability of TF-AT after fermentation increased significantly (*p* < 0.01), reaching 85.36% (take 24 h as an example), which increased for 0.93 ± 0.06 and 1.19 ± 0.12 times compared with EH-AT and AE-AT, respectively. With the progress of fermentation, a high level of free radical scavenging ability was still maintained. In addition, the ⋅OH radical scavenging ability of TF-AT-24 was comparable to the positive control ascorbic acid at 0.25 mg/mL. Consequently, different free radical scavenging assays showed different antioxidant activity, which might be due to the different binding sites in the structures of phenolic compounds, furtherly they can eliminate specific free radicals through different mechanisms, and the different chemical reactions result in differential antioxidant capacities (54, 55).

The occurrence of many chronic diseases might be related to cell, tissue, and even organ damage caused by excessive free radical production (56). Thereby, the dynamic balance between antioxidants such as phenolic compounds or flavonoids, and the generation and elimination of free radicals in the body is particularly important (57). The phenolic compounds in *A. trifoliatus* can thus be considered to have good scavenging ability for DPPH⋅ and ABTS+ free radicals (10, 12), and fermentation can thus increase the content of total phenolic compounds, thereby increasing nutritional and health benefits.

Generally, the bioactive components from plants are closely related to their antioxidant capacities. Their mechanism of antioxidants often involves hydrogen atom transfer or transfer of a single electron, among other mechanisms (58). To this end, we explored the correlations between ABTS+ values, DPPH⋅ values, and ⋅OH values with total phenolics, total flavonoids, and total polysaccharides, between AE-AT, EH-AT, and the fermentation supernatant from TF-AT, respectively (Table 4). The ABTS+ free radical scavenging activity DPPH⋅ and total phenolics showed a strong correlation (*r* = 0.984, *p* < 0.01). Additionally, there were significant correlations between DPPH⋅ free radical scavenging activity and total phenolics (*r* = 0.958, *p* < 0.01), and total flavonoids (*r* = 0.814, *p* < 0.05). Moreover, the OH free radical scavenging activity showed a significant correlation with the level of total phenolics (*r* = 0.983, *p* < 0.01) and total flavonoids (*r* = 0.933, *p* < 0.01), respectively. However, the polysaccharide content and the three free radical scavenging rates all showed insignificant positive correlations (*p* > 0.05). It is well known that DPPH⋅ and ABTS methods have free radical scavenging activities. However, these two methods revealed different free radical scavenging activities and can be used to verify the antioxidant activity of the fermentation broth from different perspectives. Table 4 shows the differences in these two testing methods. Both total phenols and total flavonoids were significantly correlated with ⋅OH free radical scavenging activity (*p* < 0.01), which seemed to indicate that ⋅OH free radical scavenging experiments were suitable as an antioxidant evaluation standard for this experimental system. Similarly, Huang et al. (59) found through Pearson correlation analysis that the total phenolic and total flavonoid content of *Rubus* fruits were significantly positively correlated with the antioxidant activity. Our results indicated that the fermentation supernatant of TF-AT had good antioxidant potential, and could be used to further explore antioxidant effects *in vivo*.

**TABLE 4:** Correlation of total phenolics, total flavonoids, total polysaccharides, and antioxidant activity among AE-AT, EH-AT, and the fermentation supernatant of TF-AT.

	Total phenolics	Total flavonoids	Total polysaccharides	ABTS+ free radical scavenging rate	DPPH⋅ free radical scavenging rate	⋅OH free radical scavenging rate
Total phenolics	1	0.868*	0.621	0.984**	0.958**	0.983**
Total flavonoids		1	0.645	0.769	0.814*	0.933**
Total polysaccharides			1	0.537	0.720	0.590
ABTS+ values				1	0.937**	0.943**
DPPH⋅ values					1	0.917**
⋅OH values						1

*Significant at < 0.05, and **significant at 0.001 < *p* < 0.01.

### Analysis of untargeted metabolomics

#### Overall analysis of metabolites in different groups

We identified different groups of metabolites based on data from ultra-high performance liquid-mass spectrometry (UPLC-MS/MS) combined with the XCMS package in R, and further explored the effects of enzymatic pre-treatment and fermentation on metabolites. By comparing with mass-to-nucleus ratios, retention times, peak areas, and other information in our database, a total of 174 phenolic compounds were preliminarily identified (Supplementary Table 1), including 108 flavonoids. Additionally, the flavonoids included 24 isoflavones and nine flavonols.

**FIGURE 5:**
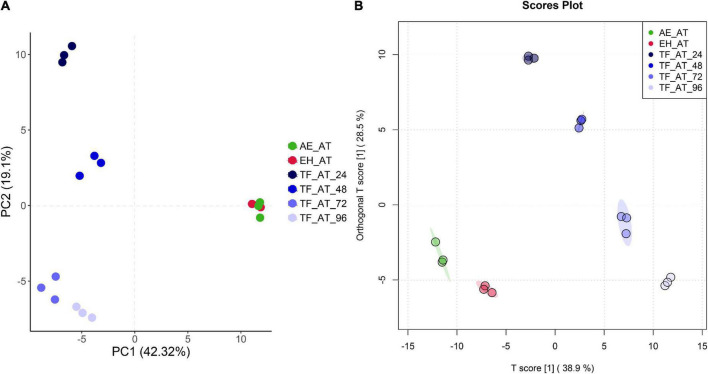
The plot of **(A)** PCA and **(B)** OPLS-DA scores for different groups. AE-AT: *aqueous extracts*–*Acanthopanax trifoliatus*; EH-AT: *enzymatic hydrolyzate*–*Acanthopanax trifoliatus;* TF-AT: fermentation supernatant of *Tremella fuciformis*–*Acanthopanax trifoliatus.* TF-AT-24, 48, 72, and 96 mean samples with related fermentation time (h).

To analyze the differences of metabolite in different groups from a macro perspective, we applied multivariate statistical techniques for analysis. The unsupervised principal component analysis (PCA) in Figure 5A shows that in the plane projection score map formed by the first principal component (PC1) and second principal component (PC2), (AE-AT and EH-AT), TF-AT-24, TF-AT-48, (TF-AT-72 and TF-AT-96) were well differentiated. There were significant differences between fermented TF-AT and unfermented AE-AT and EH-AT, whereas there was no significant difference between AE-AT and EH-AT.

In addition, we use a more precise supervised orthogonal partial least squares discriminant analysis (OPLS-DA) to improve the accuracy of our analysis. OPLS-DA filters out information that is not related to grouping (noise), which can maximize the difference between groups compared to PCA. As shown in Figure 5B, better separation was obtained between groups including (AE-AT and EH-AT). In our permutation test of OPLS-DA, Q2 and R2Y were 0.981 and 0.998, respectively, both of which were close to 1, indicating that this model was valid and the discriminative effect was good (Supplementary Table 2; Supplementary Figure 1).

#### Comprehensive analysis of differential metabolites in different groups

**FIGURE 6:**
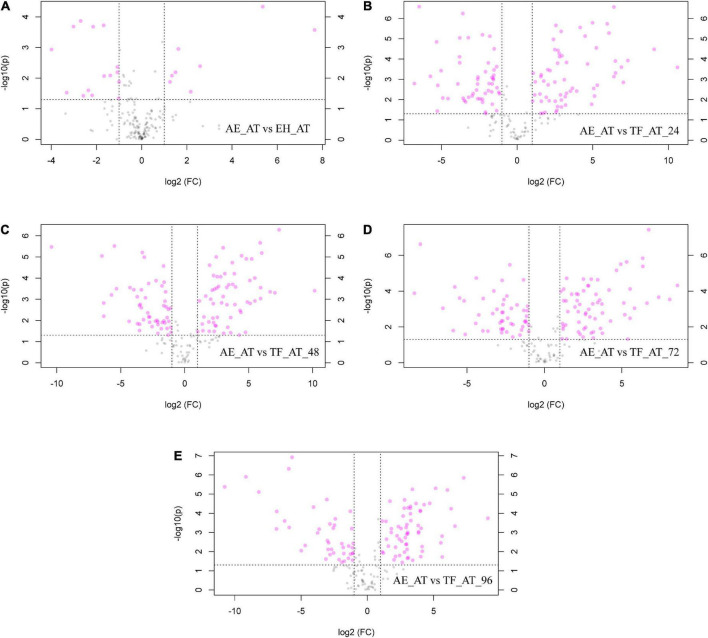
Volcano maps: **(A)** AE-AT vs. EH-AT, **(B)** AE-AT vs. TF-AT-24, **(C)** AE-AT vs. TF-AT-48, **(D)** AE-AT vs. TF-AT-72, and **(E)** AE-AT vs. TF-AT-96. The left side of the dividing line represents downregulated metabolites, and the right side represents upregulated metabolites. AE-AT: *aqueous extracts*–*Acanthopanax trifoliatus*; EH-AT: *enzymatic hydrolyzate*–*Acanthopanax trifoliatus*; TF-AT: fermentation supernatant of *Tremella fuciformis*–*Acanthopanax trifoliatus.* TF-AT-24, 48, 72, and 96 mean samples with related fermentation time (h).

To further explore the differential metabolites between groups, we created volcano plots. Each point in our volcano map (Figure 6) represents a metabolite. The abscissa represents the fold of change, and the ordinate represents the *p*-value from a *T*-test. The larger the fold change, the smaller the *p*-value and the higher the log10(*p*) was. The figure shows the number of metabolites that were significantly upregulated (log2(FC) > 1) or significantly decreased (log2(FC) < 1) between groups, while in specific the significantly different metabolite numbers of EH-AT, TF-AT-24, TF-AT-48, TF-AT-72, and TF-AT-96 compared with AE-AT were 23 (eight up, 15 down), 114 (62 up, 52 down), 114 (61 up, 53 down), 116 (65 up, 51 down), and 104 (62 up, 42 down), respectively. Overall, the effect of fermentation on metabolites was obvious, and most of the metabolites of the fermentation supernatant of TF-AT were upregulated at different times, indicating that the transformation and generation of metabolites was the main internal mechanism of the fermentation process.

**FIGURE 7:**
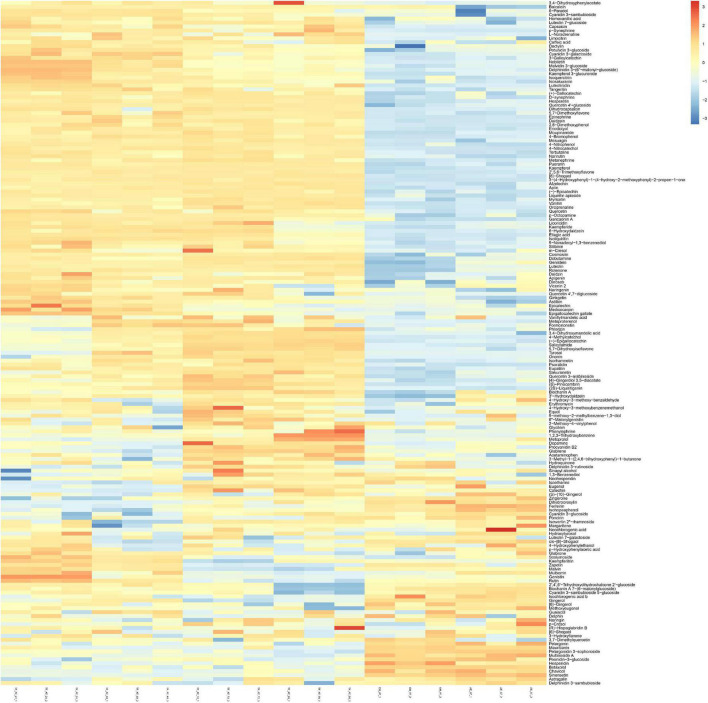
Analysis of untargeted metabolomics clustering heatmap. AE-AT: *aqueous extracts*–*Acanthopanax trifoliatus*; EH-AT: *enzymatic hydrolyzate*–*Acanthopanax trifoliatus*; TF-AT: fermentation supernatant of *Tremella fuciformis*–*Acanthopanax trifoliatus.* TF-AT-24, 48, 72, and 96 mean samples with related fermentation time (h).

In addition, we performed grouped clustering heatmap analysis (Figure 7) for 174 phenolic compounds (Supplementary Table 1). As shown in the figure, the ordinate and abscissa are the metabolites, and group names, respectively. Red to blue represents the change in metabolite abundance from high to low. Two groups of AE-AT and EH-AT were closely distributed, and the difference between these two groups was not obvious. However, there was a clear dividing line between the above two groups and the fermentation supernatant of TF-AT after 24–96 h culture. In addition, (TF-AT-24 and TF-AT-48) and (TF-AT-72 and TF-AT-96) could be clearly distinguished. In particular, more than half of the metabolites after fermentation were higher than those before fermentation.

#### Important differential metabolites in different groups

During the above supervised OPLS-DA analysis (Supplementary Figure 2), we identified the most important metabolites involved using discriminant analysis (Value Importance in Projection), namely VIP. These metabolites could then be used as an important reference to distinguish different group objects. VIP > 1 and *p* < 0.05 were differential metabolites, and the higher the VIP value, the greater the contribution to the grouping. Combined with the general table of phenolic compounds (Supplementary Table 1), we screened out the metabolites with VIP > 1 and *p* < 0.05 (Supplementary Table 3) to obtain a total of 87 phenolic compounds, including 54 flavonoids.

In past studies, the phenolic compound ellagic acid has often been studied as a strong antioxidant (60). It can be seen from Supplementary Table 3 that the ellagic acid content increased significantly after fermentation. This result may have been due to the hydrolysis of ester bonds in polymer tannins by tannase or tannyl hydrolase, so that the small molecule ellagic acid active was released (61). The content of phenolic compounds with strong antioxidant activities, such as vanillin (62, 63), increased significantly after fermentation. In addition, the content of flavonoids in metabolites, such as luteolin, kaempferol, myricetin, isorhamnetin, and (+)-gallocatechin, were significantly increased, which have all been shown to play a crucial role in the process of antioxidation and scavenging free radicals (64). The increase in the content of these compounds may have been due to the decomposition of high-polarity conjugated glycosides into low-polarity flavonoids caused by fermentation (65). The rutin content decreased with the prolongation of fermentation time. Similarly, Zhang et al. (66) found that during the fermentation of *Aspergillus niger*, the rutin content in Tartary buckwheat leaves increased in the early stages of fermentation, while then gradually decreasing.

A total of seven special glycosylated metabolites were screened for key differential metabolites (VIP > 1), namely 2′,4′,6′-trihydroxydihydrochalcone 2′-glucoside, delphinidin 3-sambubioside, pelargonidin-3-sophoroside, cyanidin 3-sambubioside 5-glucoside, luteolin 7-glucoside, peonidin-3-glucoside, and quercetin-4′-glucoside. Some fungi possess specific glycosyltransferases, which can transfer glycosidic bonds to designated metabolites, thereby increasing the diversity and stability of target metabolites (67) and improving their antioxidant capacities (68). In addition, studies have shown that the modification of glycosylation structure can improve the bioavailability of compounds like quercetin (69).

In addition, cyanidin 3-sambubioside 5-glucoside, delphinidin 3-sambubioside, and pelargonidin 3-sophoroside among the seven glycosylated metabolites, as metabolites of anthocyanins, are well known for their strong antioxidant capacities (70, 71). Interestingly, when analyzing the metabolite differences in combination with Supplementary Table 1, we found that the content of the three glycosylated flavonoids luteolin 7-glucoside, quercetin 4′,7-diglucoside, and cyanidin 3-sambubioside were slightly higher in EH-AT than in AE-AT. This may explain why the total flavonoid content of EH-AT and AE-AT was not significantly different in Figure 3B (*p* > 0.05), but the antioxidant capacity of EH-AT was stronger than AE-AT in Figure 4 (*p* < 0.05).

As is well known, fermentation is a very complex biochemical process. Not only the content of bioactive components, but also the types of bioactive components, may vary during the fermentation process (72). No significant differences in the total phenolic content were observed with a change of the pH, inoculum size or temperature in the selected range of the single-factor experiments. However, the initial pH showed a positive correlation with the total phenolic content of the fermentation supernatant in a valid response surface model. We tried to use the free-radical scavenging activity of DPPH⋅ and ABTS as a response value in our previous study; however, the model was not statistically significant (data not present). Moreover, the phenolic profile (Figure 5) and the antioxidant activity of the fermented group (TF-AT) was significantly improved compared to the two unfermented control groups of AE-AT and EH-AT (Figure 4).

Taken together, these results illustrate the complex relationship between fermentation metabolites and antioxidant activity. Additionally, fermentation processes changed the phenolic profile significantly compared with the two unfermented controls based on our untargeted metabolomics analysis (Figure 5). Similarly, a total of 25 new compounds, including six phenolic compounds, were detected in liquid fermentation of *Schizophyllum* with added *Pueraria* in our previous work (21). Zhai et al. (73) used whole grain wheat and two mushrooms for solid-state fermentation and detected a total of 15 new phenolic compounds.

We established a bidirectional fermentation system of *T. fuciformis* and *A. trifoliatus*, preliminarily indicating the chemical profiles of phenolics, flavonoids, and polysaccharides, particularly phenolics, which varied, and antioxidant capacity determined *in vitro*. However, to clarify the detailed relationship between phenolic changes (quantity and varieties) and antioxidant activity needs further study, such as fraction of the fermentation supernatant, structure identification of metabolites and targeted metabolomics analysis.

## Conclusion

In the current study, we selected the optimal fermentation conditions for *T. fuciformis* and *A. trifoliatus* (fermentation temperatures: 28°C, inoculum size: 2%, v/v, initial pH: 8) using a RSM. Under the optimal conditions, the content of total phenolics, flavonoids, and polysaccharides in the fermentation supernatant of *T. fuciformis*–*A. trifoliatus* were all significantly increased (*p* < 0.05), which increase by 0.88 ± 0.04, 0.09 ± 0.02, and 33.84 ± 1.85 times that of aqueous extracts of *A. trifoliatus*, respectively. Evaluating three different antioxidant mechanisms revealed that the fermentation supernatant from TF-AT had stronger antioxidant activity, of which the ABTS+, DPPH⋅, and ⋅OH clearance rates increased for 0.30 ± 0.001, 0.26 ± 0.01, and 1.19 ± 0.12 times compared with aqueous *A. trifoliatus* extracts, respectively. Interestingly, in our correlation analysis, phenolics from three different treatments (the aqueous extracts–*A. trifoliatus*, enzymatic hydrolyzate–*A. trifoliatus*, and the fermentation supernatant of *T. fuciformis*–*A. trifoliatus*) were found to be significantly correlated with the antioxidant capacities of ABTS+, DPPH⋅, and ⋅OH, as well as clearance rates. To further explore the endogenous mechanism leading to this phenomenon, we performed an untargeted metabolomic analysis based on UPLC-MS/MS. The results indicated that there were significant differences among the aqueous extracts–*A. trifoliatus*, enzymatic hydrolyzate–*A. trifoliatus*, and the fermentation supernatant of *T. fuciformis*–*A. trifoliatus* pre-treated with cellulase. The content of most monomeric metabolites after fermentation was higher than that before fermentation, which may be the key factor leading to the difference in antioxidant activity. The bidirectional fermentation of *T. fuciformis*–*A. trifoliatus* was shown to have good development prospects as an antioxidant and even should be involved in the research and development of functional products. To further explore the potential of the fermentation supernatant of *T. fuciformis*–*A. trifoliatus*, research on the mechanism and pharmacodynamics can be conducted in the future.

## Data availability statement

The original contributions presented in this study are included in the article/Supplementary material, further inquiries can be directed to the corresponding authors.

## Author contributions

LZ and YP: conceptualization, supporting, funding, and supervision. YW, JW, and KO: conducting experiment and data analyses. YW, YL, and JC: writing—original draft preparation. QH, TD, and LZ: review and editing. All authors have read and agreed to the published version of the manuscript.

## Supplementary material

Supplementary Figure 1 Model verification permutation test diagram of OPLS-DA. Q2 represents the predictive ability of the model.

Supplementary Figure 2 The most important metabolites involved in the OPLS-DA discriminant analysis (Value importance in projection). VIP > 1 and *p* < 0.05 are differential metabolites, and the higher the VIP value, the greater the contribution to the grouping.

## References

[1] Saoudi MM Bouajila J Alouani K. Phenolic compounds of *Rumex roseus* L. Extracts and their effect as antioxidant and cytotoxic activities. *Biomed Res Int.* (2021) 2021:2029507. 10.1155/2021/2029507 34608436 PMC8487361

[2] Vuolo MM Lima VS Junior M. Chapter 2 - phenolic compounds: structure, classification, and antioxidant power. In: Campos MRS editor. *Bioactive Compounds.* Cambridge: Woodhead Publishing (2019). p. 33–50. 10.1016/B978-0-12-814774-0.00002-5

[3] Fernandez-Panchon MS Villano D Troncoso AM Garcia-Parrilla MC. Antioxidant activity of phenolic compounds: from in vitro results to in vivo evidence. *Crit Rev Food Sci Nutr.* (2008) 48:649–71. 10.1080/10408390701761845 18663616

[4] Shalaby S Horwitz BA. Plant phenolic compounds and oxidative stress: integrated signals in fungal-plant interactions. *Curr Genet.* (2015) 61:347–57. 10.1007/s00294-014-0458-6 25407462

[5] Mittal M Siddiqui MR Tran K Reddy SP Malik AB. Reactive oxygen species in inflammation and tissue injury. *Antioxid Redox Signal.* (2014) 20:1126–67. 10.1089/ars.2012.5149 23991888 PMC3929010

[6] Rahman MM Rahaman MS Islam MR Rahman F Mithi FM Alqahtani T Role of phenolic compounds in human disease: current knowledge and future prospects. *Molecules.* (2021) 27:233. 10.3390/molecules27010233 35011465 PMC8746501

[7] Sithisarn P Jarikasem S. Antioxidant activity of *Acanthopanax trifoliatus*. *Med Princ Pract.* (2009) 18:393–8. 10.1159/000226294 19648763

[8] Li DL Zheng X Chen YC Jiang S Zhang Y Zhang WM Terpenoid composition and the anticancer activity of *Acanthopanax trifoliatus*. *Arch Pharm Res.* (2016) 39:51–8. 10.1007/s12272-015-0655-y 26345267

[9] Peng Q Chen J Duan H Wang C. Determination of kaurenoic acid in acanthopanax trifoliatus by ultra-high performance liquid chromatography coupled with tandem mass spectrometry (UHPLC-MS/MS). *Sci Rep.* (2020) 10:3378. 10.1038/s41598-020-60426-3 32099028 PMC7042316

[10] Sithisarn P Jarikasem S Thisayakorn K. Acanthopanax trifoliatus, a Potential Adaptogenic Thai Vegetable for Health Supplement[M]. In: Rasooli I editor. *Phytochemicals-Bioactivities and Impact on Health.* Rijeka, Croatia: Intech (2011).

[11] Chen Z Cheng S Lin H Wu W Liang L Chen X Antibacterial, anti-inflammatory, analgesic, and hemostatic activities of *Acanthopanax trifoliatus* (L.) merr. *Food Sci Nutr.* (2021) 9:2191–202. 10.1002/fsn3.2190 33841835 PMC8020913

[12] Wu KX Liu J Liu Y Guo XR Mu LQ Hu XH A comparative metabolomics analysis reveals the tissue-specific phenolic profiling in two *Acanthopanax* species. *Molecules.* (2018) 23:2078. 10.3390/molecules23082078 30127238 PMC6222473

[13] Ukai S Hirose K Kiho T Hara C Irikura T. Antitumor activity on sarcoma 180 of the polysaccharides from tremella fuciformis berk. *Chem Pharm Bull.* (1972) 20:2293–4. 10.1248/cpb.20.2293 4650367

[14] Xiao H Li H Wen Y Jiang D Zhu S He X Tremella fuciformis polysaccharides ameliorated ulcerative colitis via inhibiting inflammation and enhancing intestinal epithelial barrier function. *Int J Biol Macromol.* (2021) 180:633–42. 10.1016/j.ijbiomac.2021.03.083 33744251

[15] Ma X Yang M He Y Zhai C Li C. A review on the production, structure, bioactivities and applications of *Tremella* polysaccharides. *Int J Immunopathol Pharmacol.* (2021) 35:20587384211000541. 10.1177/20587384211000541 33858263 PMC8172338

[16] Yang D Liu Y Zhang L. Tremella polysaccharide: the molecular mechanisms of its drug action. *Prog Mol Biol Transl Sci.* (2019) 163:383–421. 10.1016/bs.pmbts.2019.03.002 31030755

[17] Li H Lee HS Kim SH Moon B Lee C. Antioxidant and anti−inflammatory activities of methanol extracts of *Tremella* fuciformis and its major phenolic acids. *J Food Sci.* (2014) 79:C460–8. 10.1111/1750-3841.12393 24547933

[18] Lee J Ha SJ Lee HJ Kim MJ Kim JH Kim YT Protective effect of *Tremella fuciformis* berk extract on LPS-induced acute inflammation via inhibition of the NF-κB and MAPK pathways. *Food Funct.* (2016) 7:3263–72. 10.1039/c6fo00540c 27334265

[19] Ji YW Rao GW Xie GF. Ultrasound-assisted aqueous two-phase extraction of total flavonoids from *Tremella* fuciformis and antioxidant activity of extracted flavonoids. *Prep Biochem Biotechnol.* (2022) 52:1060–8. 10.1080/10826068.2022.2028636 35098874

[20] Yi Z Xiaomei X. [Primary studies of toxicity-reducing and efficacy-maintaining action of fungal fermentative products in *Tripterygium wilfordii* by a novel bi-directional solidstate fungal fermentation]. *China J Chinese Materia Medica.* (2009) 34:2083–7. 19938552

[21] Deng Y Huang Q Hu L Liu T Zheng B Lu D Enhanced exopolysaccharide yield and antioxidant activities of schizophyllum commune fermented products by the addition of radix puerariae. *RSC Adv.* (2021) 11:38219–34. 10.1039/d1ra06314f 35498081 PMC9044015

[22] Deng Y Liu H Huang Q Tu L Hu L Zheng B Mechanism of longevity extension of caenorhabditis elegans induced by schizophyllum commune fermented supernatant with added radix puerariae. *Front Nutr.* (2022) 9:847064. 10.3389/fnut.2022.847064 35360681 PMC8963188

[23] Wang C Zhang J Hu F Zhang S Lu J Liu S. Bio-pretreatment promote hydrolysis and acidification of oilseed rape straw: roles of fermentation broth and micro-oxygen. *Bioresour Technol.* (2020) 308:123272. 10.1016/j.biortech.2020.123272 32276202

[24] Walters NA de Villiers A Joubert E de Beer D. Improved HPLC method for rooibos phenolics targeting changes due to fermentation. *J Food Comp Anal.* (2016) 55:20–9. 10.1016/j.jfca.2016.11.003

[25] Fukuta Y Shirasaka N Ikenaga C Kusuda M Yamauchi M Terashita T. Purification and characterization of endo-type cellulase of hypoxylon truncatum, a companion fungus of *Tremella* fuciformis. *Mushroom Sci Biotechnol.* (2013) 21:123–8. 10.24465/msb.21.3_123

[26] Wang HQ Li DL Lu YJ Cui XX Zhou XF Lin WP Anticancer activity of *Acanthopanax trifoliatus* (L) Merr extracts is associated with inhibition of NF-kB activity and decreased Erk1/2 and Akt phosphorylation. *Asian Pac J Cancer Prev.* (2014) 15:9341–6. 10.7314/apjcp.2014.15.21.9341 25422222

[27] Tao YM Xu XQ Ma SJ Liang G Wu XB Long MN Cellulase hydrolysis of rice straw and inactivation of endoglucanase in urea solution. *J Agric Food Chem.* (2011) 59:10971–5. 10.1021/jf203712n 21919515

[28] Darikvand F Ghavami M Honarvar M. Determination of the phenolic content in iranian trehala manna and evaluation of their antioxidant effects. *Evid Based Complement Alternat Med.* (2021) 2021:8570162. 10.1155/2021/8570162 34512783 PMC8426089

[29] Gautam VS Singh A Kumari P Nishad JH Kumar J Yadav M Phenolic and flavonoid contents and antioxidant activity of an endophytic fungus *Nigrospora sphaerica* (EHL2), inhabiting the medicinal plant *Euphorbia hirta* (dudhi) L. *Arch Microbiol.* (2022) 204:140. 10.1007/s00203-021-02650-7 35039945 PMC8763303

[30] Khatri D Chhetri SBB. Reducing sugar, total phenolic content, and antioxidant potential of nepalese plants. *Biomed Res Int.* (2020) 2020:7296859. 10.1155/2020/7296859 33274222 PMC7683130

[31] Chen Y Chen L Xiao Z Gao L. Effects of enzymolysis and fermentation on the antioxidant activity and functional components of a coarse cereal compound powder based on principal component analysis and microstructure study. *J Food Sci.* (2022) 87:3573–87. 10.1111/1750-3841.16217 35762634 PMC9544778

[32] Li W Ji J Chen X Jiang M Rui X Dong M. Structural elucidation and antioxidant activities of exopolysaccharides from *Lactobacillus helveticus* MB2-1. *Carbohydr Polym.* (2014) 102:351–9. 10.1016/j.carbpol.2013.11.053 24507291

[33] Dunn WB Broadhurst D Begley P Zelena E Francis-McIntyre S Anderson N Procedures for large-scale metabolic profiling of serum and plasma using gas chromatography and liquid chromatography coupled to mass spectrometry. *Nat Protoc.* (2011) 6:1060–83. 10.1038/nprot.2011.335 21720319

[34] Zelena E Dunn WB Broadhurst D Francis-McIntyre S Carroll KM Begley P Development of a robust and repeatable UPLC-MS method for the long-term metabolomic study of human serum. *Anal Chem.* (2009) 81:1357–64. 10.1021/ac8019366 19170513

[35] Want EJ Masson P Michopoulos F Wilson ID Theodoridis G Plumb RS Global metabolic profiling of animal and human tissues via UPLC-MS. *Nat Protoc.* (2013) 8:17–32. 10.1038/nprot.2012.135 23222455

[36] Ojo I Apiamu A Egbune EO Tonukari NJ. Biochemical characterization of solid-state fermented cassava stem (Manihot esculenta Crantz-MEC) and its application in poultry feed formulation. *Appl Biochem Biotechnol.* (2022) 194:2620–31. 10.1007/s12010-022-03871-2 35230606

[37] Zou M Zhang W Dong Q Tang C Cao F Su E. Submerged fermentation of *Ginkgo biloba* seed powder using *Eurotium cristatum* for the development of ginkgo seeds fermented products. *J Sci Food Agric.* (2021) 101:1782–91. 10.1002/jsfa.10792 32892346

[38] Ge X Huang W Xu X Lei P Sun D Xu H Production, structure, and bioactivity of polysaccharide isolated from *Tremella fuciformis* XY. *Int J Biol Macromol.* (2020) 148:173–81. 10.1016/j.ijbiomac.2020.01.021 31917978

[39] Cho EJ Oh JY Chang HY Yun JW. Production of exopolysaccharides by submerged mycelial culture of a mushroom *Tremella fuciformis*. *J Biotechnol.* (2006) 127:129–40. 10.1016/j.jbiotec.2006.06.013 16872706

[40] Robbins RJ. Phenolic acids in foods: an overview of analytical methodology. *J Agric Food Chem.* (2003) 51:2866–87. 10.1021/jf026182t 12720366

[41] Bei Q Chen G Yan L Zhang Y Wu Z. Improving phenolic compositions and bioactivity of oats by enzymatic hydrolysis and microbial fermentation. *J Func Foods.* (2018) 47:512–20. 10.1016/j.jff.2018.06.008

[42] Liu L Zhang R Deng Y Zhang Y Xiao J Huang F Fermentation and complex enzyme hydrolysis enhance total phenolics and antioxidant activity of aqueous solution from rice bran pretreated by steaming with α-amylase. *Food Chem.* (2017) 221:636–43. 10.1016/j.foodchem.2016.11.126 27979252

[43] Juan MY Chou CC. Enhancement of antioxidant activity, total phenolic and flavonoid content of black soybeans by solid state fermentation with Bacillus subtilis BCRC 14715. *Food Microbiol.* (2010) 27:586–91. 10.1016/j.fm.2009.11.002 20510775

[44] Xin CL. Review of studies on Tremella fuciformis in China [C]//. *Proceedings of the Second National Academic Exchange Conference of Young and Middle-aged Experts on Edible Fungi*, Hangzhou. Mycological Society of China. (2008). p. 18–21.

[45] Zhang J Li M Cheng J Zhang X Li K Li B Viscozyme L hydrolysis and *Lactobacillus* fermentation increase the phenolic compound content and antioxidant properties of aqueous solutions of quinoa pretreated by steaming with α-amylase. *J Food Sci.* (2021) 86:1726–36. 10.1111/1750-3841 33844283

[46] Yin YR Meng ZH Hu QW Jiang Z Xian WD Li LH The hybrid strategy of thermoactinospora rubra YIM 77501T for utilizing cellulose as a carbon source at different temperatures. *Front Microbiol.* (2017) 8:942. 10.3389/fmicb.2017.00942 28611745 PMC5447088

[47] Xiao ZP Peng ZY Peng MJ Yan WB Ouyang YZ Zhu HL. Flavonoids health benefits, and their molecular mechanism. *Mini Rev Med Chem.* (2011) 11:169–77. 10.2174/138955711794519546 21222576

[48] Wen K Fang X Yang J Yao Y Nandakumar KS Salem ML Recent research on flavonoids and their biomedical applications. *Curr Med Chem.* (2021) 28:1042–66. 10.2174/0929867327666200713184138 32660393

[49] Qiao L Sun Y Chen R Fu Y Zhang W Li X Sonochemical effects on 14 flavonoids common in citrus: relation to stability. *PLoS One.* (2014) 9:e87766. 10.1371/journal.pone.0087766 24516562 PMC3916345

[50] Dulf FV Vodnar DC Socaciu C. Effects of solid-state fermentation with two filamentous fungi on the total phenolic contents, flavonoids, antioxidant activities and lipid fractions of plum fruit (*Prunus domestica* L.) by-products. *Food Chem.* (2016) 209:27–36. 10.1016/j.foodchem.2016.04.016 27173530

[51] Septembre-Malaterre A Remize F Poucheret P. Fruits, and vegetables, as a source of nutritional compounds and phytochemicals: changes in bioactive compounds during lactic fermentation. *Food Res Int.* (2018) 104:86–99. 10.1016/j.foodres.2017.09.031 29433787

[52] Filannino P Di Cagno R Gobbetti M. Metabolic and functional paths of lactic acid bacteria in plant foods: get out of the labyrinth. *Curr Opin Biotechnol.* (2018) 49:64–72. 10.1016/j.copbio.2017.07.016 28830019

[53] Zhao YS Eweys AS Zhang JY Zhu Y Bai J Darwesh OM Fermentation affects the antioxidant activity of plant-based food material through the release and production of bioactive components. *Antioxidants.* (2021) 10:2004. 10.3390/antiox10122004 34943107 PMC8698425

[54] Fernández-Moriano C González-Burgos E Divakar PK Crespo A Gómez-Serranillos MP. Evaluation of the antioxidant capacities and cytotoxic effects of ten parmeliaceae lichen species. *Evid Based Complement Alternat Med.* (2016) 2016:3169751. 10.1155/2016/3169751 28074101 PMC5203883

[55] Kwaw E Ma Y Tchabo W Apaliya MT Wu M Sackey AS Effect of lactobacillus strains on phenolic profile, color attributes and antioxidant activities of lactic-acid-fermented mulberry juice. *Food Chem.* (2018) 250:148–54. 10.1016/j.foodchem.2018.01.009 29412905

[56] Hassan HA Ghareb NE Azhari GF. Antioxidant activity and free radical-scavenging of cape gooseberry (*Physalis peruviana* L.) in hepatocellular carcinoma rats model. *Hepatoma Res.* (2017) 3:27–33. 10.20517/2394-5079.2016.33

[57] Freire AL Ramos CL da Costa Souza PN Cardoso MG Schwan RF. Nondairy beverage produced by controlled fermentation with potential probiotic starter cultures of lactic acid bacteria and yeast. *Int J Food Microbiol.* (2017) 248:39–46. 10.1016/j.ijfoodmicro.2017.02.011 28242421

[58] Zeb A. Concept, mechanism, and applications of phenolic antioxidants in foods. *J Food Biochem.* (2020) 44:e13394. 10.1111/jfbc.13394 32691460

[59] Huang X Wu Y Zhang S Yang H Wu W Lyu L Variation in bioactive compounds and antioxidant activity of *Rubus* fruits at different developmental stages. *Foods.* (2022) 11:1169. 10.3390/foods11081169 35454756 PMC9026527

[60] Kilic I Yeşiloğlu Y Bayrak Y. Spectroscopic studies on the antioxidant activity of ellagic acid. *Spectrochim Acta A Mol Biomol Spectrosc.* (2014) 130:447–52. 10.1016/j.saa.2014.04.052 24813273

[61] Curiel JA Pinto D Marzani B Filannino P Farris GA Gobbetti M Lactic acid fermentation as a tool to enhance the antioxidant properties of *Myrtus* communis berries. *Microb Cell Fact.* (2015) 14:67. 10.1186/s12934-015-0250-4 25947251 PMC4424524

[62] Tai A Sawano T Yazama F Ito H. Evaluation of antioxidant activity of vanillin by using multiple antioxidant assays. *Biochim Biophys Acta.* (2011) 1810:170–7. 10.1016/j.bbagen.2010.11.004 21095222

[63] Mao QQ Xu XY Cao SY Gan RY Corke H Beta T Bioactive compounds and bioactivities of Ginger (*Zingiber officinale Roscoe*). *Foods.* (2019) 8:185. 10.3390/foods8060185 31151279 PMC6616534

[64] Griffiths K Aggarwal BB Singh RB Buttar HS Wilson D De Meester F. Food antioxidants and their anti-inflammatory properties: a potential role in cardiovascular diseases and cancer prevention. *Diseases.* (2016) 4:28. 10.3390/diseases4030028 28933408 PMC5456284

[65] Shang Z Li M Zhang W Cai S Hu X Yi J. Analysis of phenolic compounds in pickled chayote and their effects on antioxidant activities and cell protection. *Food Res Int.* (2022) 157:111325. 10.1016/j.foodres.2022.111325 35761610

[66] Zhang XY Chen J Li XL Ye Y Wang SF Hu HL Dynamic changes in antioxidant activity and biochemical composition of tartary buckwheat leaves during Aspergillus niger fermentation. *J Func Foods.* (2017) 32:375–81. 10.1016/j.jff.2017.03.022

[67] Wang H Wang C Fan W Yang J Appelhagen I Wu Y A novel glycosyltransferase catalyses the transfer of glucose to glucosylated anthocyanins in purple sweet potato. *J Exp Bot.* (2018) 69:5444–59. 10.1093/jxb/ery305 30124996 PMC6255700

[68] Zhao Z Wu X Chen H Liu Y Xiao Y Chen H Evaluation of a strawberry fermented beverage with potential health benefits. *PeerJ.* (2021) 9:e11974. 10.7717/peerj.11974 34513326 PMC8388556

[69] Shi J Yang G You Q Sun S Chen R Lin Z Updates on the chemistry, processing characteristics, and utilization of tea flavonoids in last two decades (2001-2021). *Crit Rev Food Sci Nutr.* (2021):1–28. [Epub ahead of print]. 10.1080/10408398.2021.2007353 34898343

[70] Mandrone M Lorenzi B Maggio A La Mantia T Scordino M Bruno M Polyphenols pattern and correlation with antioxidant activities of berries extracts from four different populations of Sicilian *Sambucus nigra* L. *Nat Prod Res.* (2014) 28:1246–53. 10.1080/14786419.2014.898147 24666289

[71] Winter AN Bickford PC. Anthocyanins and their metabolites as therapeutic agents for neurodegenerative disease. *Antioxidants.* (2019) 8:333. 10.3390/antiox8090333 31443476 PMC6770078

[72] Leonard W Zhang P Ying D Adhikari B Fang Z. Fermentation transforms the phenolic profiles and bioactivities of plant-based foods. *Biotechnol Adv.* (2021) 49:107763. 10.1016/j.biotechadv.2021.107763 33961978

[73] Zhai FH Chen YF Zhang Y Zhao WJ Han JR. Phenolic compounds and antioxidant properties of wheat fermented with *Agaricus brasiliensis* and *Agaricus bisporus*. *FEMS Microbiol Lett.* (2021) 368:fnaa213. 10.1093/femsle/fnaa213 33338214

