# Supplementary material for: Chemical profile and antioxidant activity of bidirectional metabolites from Tremella fuciformis and Acanthopanax trifoliatus as assessed using response surface methodology
Source: Front Nutr. 2022 Nov 8;9:1035788. doi: 10.3389/fnut.2022.1035788 (PMC9679022; doi:10.3389/fnut.2022.1035788)
Supplement: Supplementary file 2 [file Table_2.DOCX]

|  | R2X | R2X(cum) | R2Y | R2Y(cum) | Q2 | Q2(cum) |
| --- | --- | --- | --- | --- | --- | --- |
| p1 | 0.389 | 0.389 | 0.816 | 0.816 | 0.779 | 0.779 |
| o1 | 0.285 | 0.674 | 0.161 | 0.161 | 0.175 | 0.175 |
| o2 | 0.0657 | 0.739 | 0.021 | 0.182 | 0.0281 | 0.203 |
| sum | NA | 0.739 | NA | 0.998 | NA | 0.981 |

**Supplement Table S2.** Model verification permutation Test diagram of OPLS–DA

Explained variation R2X and R2Y are computed from the sum of squares of the X and Y matrices of the built model, respectively. Q2 represents the predictive ability of the model.
