# Supplementary figures and images for: Chemical profile and antioxidant activity of bidirectional metabolites from Tremella fuciformis and Acanthopanax trifoliatus as assessed using response surface methodology
Source: Front Nutr. 2022 Nov 8;9:1035788. doi: 10.3389/fnut.2022.1035788 (PMC9679022; doi:10.3389/fnut.2022.1035788)

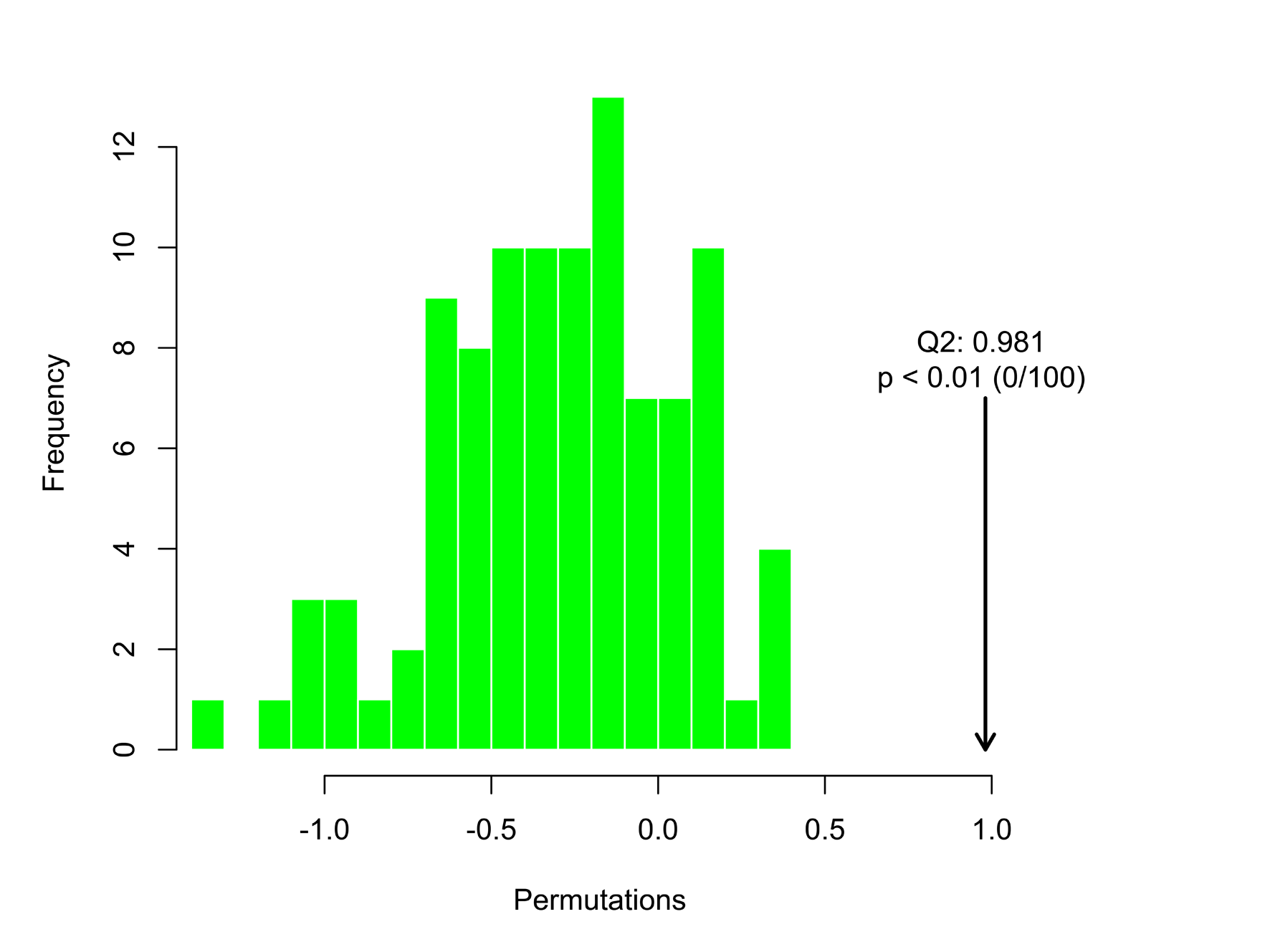

Supplement: Supplementary Figure 1 — Model verification permutation test diagram of OPLS-DA. Q2 represents the predictive ability of the model. [file Image_1.TIF]

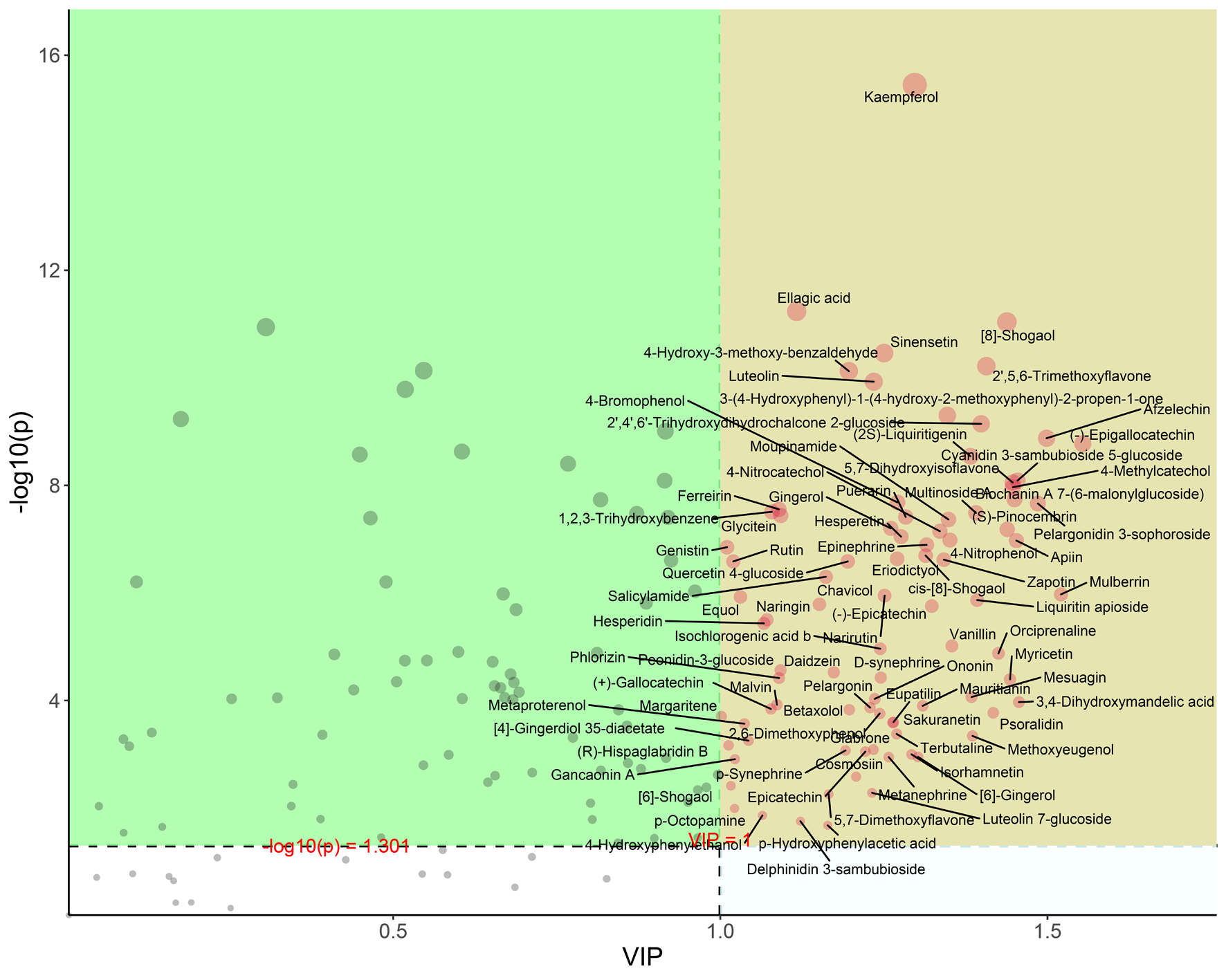

Supplement: Supplementary Figure 2 — The most important metabolites involved in the OPLS-DA discriminant analysis (Value importance in projection). VIP > 1 and p < 0.05 are differential metabolites, and the higher the VIP value, the greater the contribution to the grouping. [file Image_2.TIF]
